# A human monocytic NF-κB fluorescent reporter cell line for detection of microbial contaminants in biological samples

**DOI:** 10.1371/journal.pone.0178220

**Published:** 2017-05-24

**Authors:** Claire Battin, Annika Hennig, Patrick Mayrhofer, Renate Kunert, Gerhard J. Zlabinger, Peter Steinberger, Wolfgang Paster

**Affiliations:** 1 Division of Immune Receptors and T Cell Activation, Institute of Immunology, Center for Pathophysiology, Infectiology and Immunology, Medical University of Vienna, Vienna, Austria; 2 Department of Biotechnology, University of Natural Resources and Life Sciences, Vienna, Austria; 3 Division of Clinical and Experimental Immunology, Institute of Immunology, Center for Pathophysiology, Infectiology and Immunology, Medical University of Vienna, Vienna, Austria; Katholieke Universiteit Leuven Rega Institute for Medical Research, BELGIUM

## Abstract

Sensing of pathogens by innate immune cells is essential for the initiation of appropriate immune responses. Toll-like receptors (TLRs), which are highly sensitive for various structurally and evolutionary conserved molecules derived from microbes have a prominent role in this process. TLR engagement results in the activation of the transcription factor NF-κB, which induces the expression of cytokines and other inflammatory mediators. The exquisite sensitivity of TLR signalling can be exploited for the detection of bacteria and microbial contaminants in tissue cultures and in protein preparations. Here we describe a cellular reporter system for the detection of TLR ligands in biological samples. The well-characterized human monocytic THP-1 cell line was chosen as host for an NF-ᴋB-inducible enhanced green fluorescent protein reporter gene. We studied the sensitivity of the resultant reporter cells for a variety of microbial components and observed a strong reactivity towards TLR1/2 and TLR2/6 ligands. Mycoplasma lipoproteins are potent TLR2/6 agonists and we demonstrate that our reporter cells can be used as reliable and robust detection system for mycoplasma contaminations in cell cultures. In addition, a TLR4-sensitive subline of our reporters was engineered, and probed with recombinant proteins expressed in different host systems. Bacterially expressed but not mammalian expressed proteins induced strong reporter activity. We also tested proteins expressed in an *E*. *coli* strain engineered to lack TLR4 agonists. Such preparations also induced reporter activation in THP-1 cells highlighting the importance of testing recombinant protein preparations for microbial contaminations beyond endotoxins. Our results demonstrate the usefulness of monocytic reporter cells for high-throughput screening for microbial contaminations in diverse biological samples, including tissue culture supernatants and recombinant protein preparations. Fluorescent reporter assays can be measured on standard flow cytometers and in contrast to established detection methods, like luciferase-based systems or Limulus Amebocyte Lysate tests, they do not require costly reagents.

## Introduction

A recurrent problem in biomedical research is the presence of microbial contaminants in biological samples. Prominent and widespread examples are mycoplasma infestations of long-term cell cultures or presence of gram-negative endotoxins in recombinant proteins. Unchecked contaminations with bacterial products seriously impact on experimental research and can render data unusable. Sensitive detection methods for the presence of microbial products are therefore of vital importance. Various test systems are currently in routine use: The Limulus amebocyte lysate (LAL) test for endotoxin and various PCR-based or enzymatic tests for mycoplasma detection [1, 2]. Most of these assays are time intensive and require additional non-standard reagents and equipment. For the current study we aimed to exploit the exquisite sensitivity of evolutionary conserved pattern recognition receptors (PRRs) for the generation of a sensitive cellular reporter platform.

PRRs enable innate cells to recognize molecular structures conserved across microbial species, also known as pathogen-associated molecular patterns (PAMPs). As such, they are a crucial component of the first-line defence mechanisms following barrier breach by microbes. Additionally, several PRRs can initiate sterile inflammation by responding to endogenous danger signals, or damage-associated molecular patterns (DAMPs), released by damaged or dying cells. Currently four classes of PRRs are known: The transmembrane Toll-like receptors (TLRs), the C-type lectin receptors (CLRs), the cytoplasmic retinoic acid-inducible gene (RIG)-I-like receptors (RLRs) and NOD-like receptors (NLRs). Toll was discovered two decades ago as an essential receptor in anti-fungal host defence in *Drosophila*, which lacks adaptive immunity [3–5]. Shortly afterwards, TLR4 was described as the first mammalian homolog of Toll, driving NF-κB-mediated expression of inflammatory cytokines in response to lipopolysaccharide (LPS) [5]. TLRs are type I integral membrane glycoproteins with an N-terminal ligand recognition domain, a single transmembrane helix, and a C-terminal cytoplasmic signalling domain. The N-terminal TLR ectodomains (ECDs) encounter microbial components either at the exterior cell surface or intracellularly in the lumen of the endosomal/lysosomal compartment. TLR-ECDs are composed of 19–25 leucine-rich repeats (LRR), giving rise to the prototypic large horseshoe shape. The C-terminal signalling domains are known as Toll/IL-1 receptor (TIR) domains because they share homology with the signalling domains of IL-1 receptor family members [6, 7]. Up to date ten TLR genes in humans have been identified, yet the ligand of TLR10 is still unknown [8, 9]. PAMPs recognized by different TLRs include bacterial cell wall components, bacterial DNA and viral double stranded RNA molecules [10–12]. The nucleotide-sensing TLRs 3, 7, 8 and 9 are localized at intracellular membranes which is in contrast to the plasma membrane where most microbial cell wall reactive TLRs reside. Toll-like receptor 2 (TLR2) has been shown to play a crucial role in recognizing peptidoglycans and lipopeptides [13, 14]. Heterodimerization with TLR6 confers specificity towards diacylated lipopeptides derived from mycoplasma, whereas TLR1/2 heterodimers react towards triacylated lipopeptides of gram-negative origin [13, 15]. LPS is the major component and virulence factor of the outer membrane of gram-negative bacteria and triggers TLR4 in complex with CD14 and MD2 [16]. Several different lipopeptides commonly associated with LPS can in addition trigger TLR2 responses [17]. Thus each individual TLR plays a specialized role in detecting microbial components and thereby contributes to the shaping of an appropriate immune response against pathogens [18]. In addition, presence of TLR ligands even at very low doses results in non-resolving inflammatory responses and might play a role in chronic diseases like atherosclerosis [19, 20]. Upon ligand engagement, TLRs form homo- or heterodimers and relay signals via two major downstream TIR-domain containing cytoplasmic adaptor proteins, myeloid differentiation primary-response protein 88 (MyD88) and TIR domain-containing adaptor protein inducing IFN-β (TRIF). While MyD88 is utilized by all TLRs, TRIF mediates an alternative pathway downstream of TLR3 and TLR4. The MyD88-dependent signalling cascade is further propagated by the engagement of members of the IRAK (IL-1R-associated kinase) serine/threonine kinases and the adaptor molecules TNF receptor-associated factors (TRAFs). These complexes induce the activation of transcription factors like nuclear factor-ᴋB (NF-ᴋB), activator protein 1 (AP-1) and the interferon-regulatory factors (IRFs) and subsequently the release of pro- and anti-inflammatory cytokines [21, 22].

Here we describe the generation of a transcriptional fluorescent reporter system, based on the human monocytic THP-1 cell line. THP-1 cells naturally express a broad range of PRRs and are commonly used to explore TLR signalling or inflammasome activation [23]. A reporter cassette comprising of an NF-κB-driven enhanced green fluorescent protein (eGFP) gene was introduced into wild-type THP-1 cells. In their basic configuration, our THP-1 NF-ᴋB-eGFP cells displayed a high and strictly dose-dependent sensitivity towards TLR2 ligands. We could demonstrate that our reporters detected mycoplasma contaminations from fresh, heat-inactivated and cryo-stored cell culture supernatants with equally high sensitivity. By introducing a combination of TLR4, CD14 and MD2, we widened the detection range towards TLR4 ligands, and successfully applied the reporters to the detection of endotoxin in recombinant protein preparations. Our novel THP-1-based reporter cell line thus represents a reliable and cost-effective tool to monitor microbial contaminations in a high-throughput format that can be conveniently analysed on standard flow cytometers.

## Materials and methods

### Reagents and cell culture

The human monocytic cell line THP-1 [24], the human myelogenous leukaemia cell line K562 [25] and HEK293 hTLR4A-MD2-CD14 (Invivogen, San Diego, CA) were maintained in RPMI 1640 supplemented with 10% heat-inactivated fetal calf serum (FCS), 100 μg/mL streptomycin and 100 U/mL penicillin. Cells were cultured in a humidified atmosphere (5% CO_2_) at 37°C. All materials were obtained from Sigma-Aldrich (St. Louis, MO), unless stated otherwise. Agonists for TLR1/2 (Pam3CSK4, synthetic triacylated lipopeptide), TLR2/6 (FSL-1, synthetic diacylated lipopeptide), TLR3 (poly I: C, synthetic analogue of double-stranded RNA (dsRNA)), TLR4 (LPS-EB ultrapure), TLR5 (Flagellin), TLR7/8 (resiquimod (R848), imidazoquinoline analogue), TLR9 (Class B CpG oligonucleotide ODN 2006) and neutralizing TLR6 mAb clone C5C8 were purchased from Invivogen (San Diego, CA). The TLR2/6 agonist MALP-2 was purchased from Novus Biologicals (Littleton, CO). Standard LPS (*Escherichia coli* 0127:B8), Phorbol-12-myristat-13-acetat (PMA) and ionomycin were obtained from Sigma Aldrich (St. Louis, MO). For mycoplasma detection experiments different potentially contaminated cell sources were provided by Maria Eisenbauer (Institute of Cancer Research, Medical University of Vienna, Austria). These cells originated from (1) mouse tail cells, (2) human mesotheliom, (3) human melanoma brain metastasis-derived cell line YDFR, (4) human LN229 glioblastoma, (5) human ovarian cancer cells, (6) COS-7 cell line and (7) human skin fibroblasts (S1 Table). Recombinant TNF-α protein was purchased from Peprotech (London, UK) and monoclonal TNF-α blocking antibody Adalimumab (trade name *Humira*) was obtained from AbbVie Inc., North Chicago, IL.

### Flow cytometry

Acquisition of flow cytometry data was performed using FACS Calibur with CellQuest software (both BD Biosciences, San Jose, CA). Data was analysed using FlowJo software (version 10.0.8., Tree Star, Ashland, OR). Fluorescence intensity is shown on a standard logarithmic scale.

### Generation of stable THP-1 NF-κB-eGFP and THP-1 TLR4-CD14 NF-κB-eGFP reporter cells

The NF-κB-eGFP reporter construct was previously described [26]. The THP-1 cells were retrovirally transduced with the NF-κB-eGFP construct and resting cells were sorted to eGFP-low expression. From these, single cell clones were established to obtain a stable expressing NF-κB-eGFP reporter cell line (S1 Fig). To obtain TLR4-sensitive reporter cells, retroviral expression constructs encoding human TLR4, CD14 and MD2 were cloned into the retroviral vector pCJK2 generated in our laboratory [27, 28]. The previously generated THP-1 NF-κB-eGFP reporter cell line was transduced with these expression vectors and single cell clones were established.

### THP-1 reporter assays

For reporter assays, THP-1 NF-κB-eGFP cells were incubated in the presence of stimuli for 24 h. Assays were performed in 96-well flat bottom plates at 5x10^4^ cells per well in a total volume of 100 μl (including stimulus). Cells were then harvested and eGFP expression was analysed by flow cytometry. Mean and standard deviation of the geometric mean of fluorescence intensity (gMFI) of the viable population of reporter cells was determined. All samples were analysed in triplicates, unless indicated otherwise. For TNF-α blocking assays, THP-1 reporter cells were incubated together with a monoclonal TNF-α antibody Adalimumab (Humira; 10 μg/ml) and different concentrations of LPS, TNF-α or mycoplasma supernatants. For TLR6 blocking experiments, THP-1 reporter cells were pre-treated with 5 μg/ml mouse IgG1 (MOPC-21) or TLR6 mAb (C5C8) for 30 min at 37°C, then stimulated with FSL-1, MALP-2, LPS, or mycoplasma supernatants. For mycoplasma detection, 50 μl cell-free tissue culture supernatants were applied to THP-1 reporters in 50 μl fresh medium (final dilution 1:2). In some experiments frozen (-20°C for at least 1 h) or heat-treated (5 min 95°C) cell culture supernatants were used. Cell cultures whose supernatants induced eGFP gMFI values in the reporter cells that were 50% higher compared to untreated reporter cells were scored as mycoplasma positive.

### Maturation of monocyte-derived dendritic cells by TLR agonists

Peripheral blood mononuclear cells (PBMCs) were isolated from heparinized whole blood of healthy volunteer donors (red-cross Austria) by standard density-gradient centrifugation with Lymphoprep (Axis-Shield PoC AS, Oslo, Norway). Donors gave their informed consent and approval was obtained from the ethics committee of the Medical University of Vienna (ECS1183/2016). Monocytes were isolated from PBMCs with the MagniSort® Human CD14 Positive Selection Kit (Thermo Fisher Scientific, Waltham, MA). Monocyte-derived dendritic cells (moDCs) were generated from the isolated monocytes by incubation with IL-4 and GM-CSF (Peprotech, London, UK) as previously described [29]. Immature moDCs (1x10^6^/well) were either left untreated or stimulated with TLR ligands in a 24-well plate (Pam3CSK4 (100 nM), FSL-1 (100 nM), MALP-2 (46,8 nM), Poly I: C (10 μg/ml), standard LPS (300 ng/ml), LPS (UP) (300 ng/ml), flagellin (100 ng/ml), imidazoquinoline (10 μg/ml) and CpG ODN 2006 (10 μg/ml). After 24 h, cell surface expression of maturation markers was assessed by flow cytometry. PE-labeled-CD83 (HB15e) and the appropriate labelled-Ab-isotype controls (MOPC-21) were purchased from Biolegend (San Diego, CA). APC-labeled-CD86 (IT2.2) was purchased from BD Bioscience (San Jose, CA).

### Monitoring mycoplasma removal

Mycoplasma negative tested K562 cells were deliberately infected with supernatant containing mycoplasma in a 24 well plate. A fully established infection was verified 7 days post infection in K562 supernatants using both THP-1 reporter cells and the MycoAlert mycoplasma detection kit (Lonza Verviers, Belgium), according to the manufacturer´s instructions. The MycoAlert kit is based on a bioluminescent reaction and detects mycoplasma enzyme activities. Following infection, four commercially available mycoplasma removal reagents were applied on the mycoplasma contaminated K562 cells according to the manufacturer´s instructions: Plasmocure (Agent 1), Plasmocin (Agent 2) (both Invivogen, San Diego, CA), BM-cyclin (Agent 3) (Sigma-Aldrich, St. Louis, MO) and Biotool mycoplasma removal kit (Agent 4) (Biotool, Houston, TX). In all cases cells were treated for three weeks. Supernatants were collected at day 2, 4, 7, 10, 15, 17, 21, 23, 25 and 29 and frozen at -20°C for further testing until the end of the treatment. Afterwards, THP-1 reporter cells (5x10^4^/well) were incubated with the collected supernatants for 24 h followed by FACS analysis. At day 29 samples were additionally tested with the MycoAlert mycoplasma detection kit. For the MycoAlert kit only fresh supernatants at the day of collection were used.

### Bacterial protein expression

A construct encoding human complement split product C4dg fused to a C-terminal 6xHIS Tag was cloned into the IPTG-inducible bacterial expression vector pET21a(+) (EMD Millipore, Billerica, MA). The electrocompetent E. coli protein expression strain ClearColi BL21 was purchased from Lucigen (Middleton, WI) and standard E. coli BL21 were obtained from New England Biolabs (Ipswich, MA). Protein expression was performed in standard LB (for BL21) or LB-Miller (10 g/L NaCl, for ClearColi BL21). Expression cultures of 1L were inoculated from overnight cultures derived from single colonies and grown to an OD_600_ of 0.6 at 37°C. Cultures were induced with 1 mM IPTG and grown for 4 h at 37°C. Bacterial pellets were washed once in ice-cold Bacterial Resuspension Buffer (50 mM Tris-HCl pH8.0, 25% Sucrose, 5 mM MgCl_2_) and stored at -80°C. Pellets were resuspended in 3 ml/gram wet pellet of Bacterial Lysis Buffer (50 mM Na-Phosphate pH8.0, 150 mM NaCl, 5% Glycerol, protease inhibitors, 2 mg/ml Lysozyme) and incubated at 37°C for 30 min followed by three freeze/thaw cycles. DNase I was added at 1 mg/ml and samples were incubated at 37°C for 15 min followed by centrifugation at 20,000xg for 20 min and filtration through a 0.45 μm syringe filter. All following protein purification steps were performed at 4°C. 1 ml HisTALON Superflow Cartridges (Clontech Laboratories Inc, Mountain View, CA) were equilibrated with Running Buffer (50 mM Na-Phosphate pH8.0, 150 mM NaCl) using a peristaltic pump. Cleared samples were passed over the column twice, followed by extensive washing with Running Buffer overnight. Purified protein was eluted in Na-Phosphate pH7.4, 150 mM NaCl, 100 mM Imidazole. Fractions of 0.5 ml were collected and analysed by SDS-PAGE and Coomassie staining. Pure, high-protein fractions were pooled and dialyzed against Na-Phosphate pH7.4, 150 mM NaCl for Imidazole removal.

### Mammalian protein expression

A construct encoding human complement split product C4dg fused to an N-terminal human CD5 signal peptide for secretion and a C-terminal 6xHIS Tag was cloned into the mammalian expression vector pCEP4 (Thermo Fisher Scientific, Waltham, MA). Transient expression was performed under serum-free conditions in suspension HEK293-6E as described by Margreitter et al. [30]. Briefly, transfection of the pCEP4 vector was performed with polyethylemine (PEI; linear 25 kDA, Polysciences) at a cell density of 1x10^6^/ml. At Day 7, supernatants were filtered through a 0.45 μm syringe filter and dialyzed extensively against 50 mM Na-Phosphate pH8.0, 150 mM NaCl prior to HisTALON IMAC purification (see above).

### Endotoxin removal

Affi-Prep Polymyxin Resin (Bio-Rad Laboratories, Hercules, CA) was washed twice with endotoxin-free PBS. Bacterial protein preparations were treated by bulk-purification under constant agitation at room temperature for 8 h at a concentration of 1 mg total protein in 1 ml with 200 μl pre-washed polymyxin resin beads.

### Quantitative PCR

For quantitative real-time PCR (qPCR) analysis, RNA was isolated using TRIzol reagent (Peqlab, Erlangen, Germany) and transcribed with the RevertAid H Minus Reverse Transcriptase (Thermo Fisher Scientific, Waltham, MA). Accumulation of PCR products was detected by monitoring the increase in fluorescence of the reporter dye SYBR Green I on a CFX96 Real-Time PCR System (both Bio-Rad Laboratories, Hercules, CA). The following primer sets were used for qPCR reactions: hTLR4 (forward: 5´-CTCTCCTGCGTGAGACCAG-3´, reverse: 5´-TCCATGCATTGATAAGTAATATTAGGA-3´), CD14 (forward: 5´-ACGCCAGAACCTTGTGAGC-3´, reverse: 5´-GCATGGATCTCCACCTCTACTG-3´) and MD2 (forward: 5´-CCGAGGATCTGATGACGATT-3´, reverse: 5´-TGGGCTCCCAGAAATAGCTT-3´).

### Microscopy

For image acquisition, 5x10^4^ THP-1 NF-κB-eGFP reporter cells were stimulated with standard LPS (3 μg/ml) and mycoplasma containing cell culture supernatants (1/10; 1/100; 1/1000 dilutions) for 24 h in a 96-well plate. Unstimulated and stimulated THP-1 NF-κB-eGFP reporter cells were directly recorded in cell culture medium. Image acquisition was performed on a Leica DMI4000 B light microscope using a 63x dry objective (Leica Microsystems, Wetzlar, Germany) and an Andor iXon Ultra-897 EM-CCD camera (Andor Technologies, Belfast, UK). The system was controlled by MetaMorph software (Molceular Devices, Downingtown, PA). Images were processed with the software ImageJ (Version 1.49, National Institute of Health, Washington, DC).

## Results

### THP-1 NF-κB-eGFP cells exhibit selective sensitivity towards TLR ligands

We have previously described the generation of Jurkat reporter cell lines based on a set of highly sensitive and selective fluorescent transcriptional reporter constructs for the activity of the transcription factors AP-1, NFAT and NF-κB [26, 31]. For the current study we aimed to employ our reporter technology for the development of a test system for ligands to toll-like receptors. We chose the human acute monocytic leukaemia cell line THP-1 for this purpose. THP-1 cells are a widely used model for monocyte/macrophage function [23, 32], expresses mRNAs encoding all known human toll-like receptors (TLR1-10) and were described to be highly sensitive to microbial components [23, 33]. A retroviral NF-κB-driven eGFP reporter construct was introduced into THP-1 cells and single cell clones were established by limiting dilution culturing. A cell clone that was negative in a resting state and strongly expressed the reporter gene upon activation with PMA and Ionomycin was selected from a large pool of clones for further analysis (S1A and S1B Fig).

**Fig 1:**
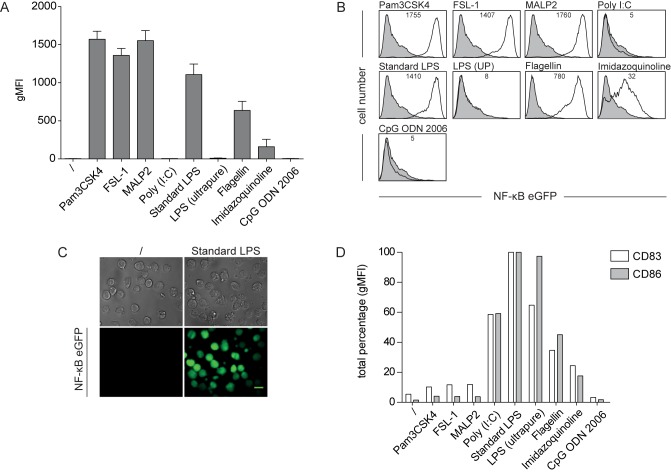
THP-1 NF-κB-eGFP reporter cells show a selective sensitivity towards TLR ligands. (A) THP-1 NF-κB-eGFP cells were incubated with Pam3CSK4 (TLR1/2; 100 nM), FSL-1 (TLR2/6; 100 nM), MALP-2 (TLR2/6; 46,8 nM), Poly I:C (TLR3; 10 μg/ml), standard LPS (TLR2/4; 300 ng/ml), LPS ultrapure (UP) (TLR4; 300ng/ml), flagellin (TLR5; 100 ng/ml), imidazoquinoline (TLR7/8; 10 μg/ml) and CpG ODN 2006 (TLR9; 50 μg/ml). After 24 h, eGFP expression was assessed by flow cytometry. Bar graphs show geometric mean of fluorescence intensity (gMFI). Mean and SE were calculated from triplicates of five independently performed experiments (n = 5). (B) Representative flow cytometry histograms of reporter gene expression in THP-1 NF-κB-eGFP cells as described in A. Open histograms: TLR-activated reporter cells; filled histograms: unstimulated reporter cells. Numbers show gMFI. (C) Fluorescent microscopy images of reporter cells activated with standard LPS (3 μg/ml) for 24 h (right panel). Unstimulated cells served as negative control (left panel). Bright field images are shown for comparison (top row). Scale bar: 10 μm. (D) Immature human monocyte-derived DCs were incubated with various TLR ligands (used at the same concentrations as in A) for 24 h or were left untreated. Expression of maturation markers CD83 and CD86 was assessed by flow cytometry. Bar graphs show total percentage of gMFI normalized to standard LPS.

In a first set of experiments, we assessed the reactivity of our THP-1 reporter cells towards TLR ligands. Cells were exposed to various TLR stimuli for 24 h and reporter gene expression was measured by flow cytometry. Treatment with Pam3CSK4, FSL-1 and MALP-2 induced a strong activation of the reporter gene, whereas flagellin and imidazoquinoline elicited lower responses. No response to Poly I:C and the class B CpG oligonucleotide ODN 2006 was detected. Interestingly, we found that although the addition of a standard LPS preparation resulted in a dramatic induction of eGFP, which could readily be observed by standard fluorescent microscopy, our THP-1 reporter cells did not respond to highly pure LPS (Fig 1A–1C). It has previously been shown that standard LPS preparations contain lipoproteins and that these impurities are potent TLR2 agonists [17, 34]. This suggests that lipoprotein contaminations rather than LPS itself mediate activation of our THP-1 reporter cells by standard LPS preparations. PAMP-sensing by dendritic cells (DCs) plays a central role in the initiation of immune responses and DCs express a distinct repertoire of TLRs [35, 36]. Consequently, we assessed the response of human monocyte-derived DCs (moDCs) to our set of TLR ligands. Up-regulation of the maturation markers CD83 and CD86 was used as a read-out for moDC-activation. We observed that except for flagellin and imidazoquinoline that acted as weak stimuli for both cell types, our THP-1 reporters and moDCs had a quite distinct reactivity profile towards TLR ligands (Fig 1D). The weak stimulation of moDCs by Pam3CSK4, FSL-1, and MALP-2 indicates a poor reactivity towards ligands for TLR2 heterodimers. In contrast to our THP-1 reporters, moDCs were strongly responding to both standard and highly pure LPS. Like THP-1 cells, moDCs did not react towards CpG ODN 2006. This is in line with previous observations and can be attributed to low levels of TLR9 expression in human moDCs and THP-1 cells [37, 38].

### Dose-dependent transcriptional activation of THP-1 NF-κB-eGFP reporters to specific TLR ligands

**Fig 2:**
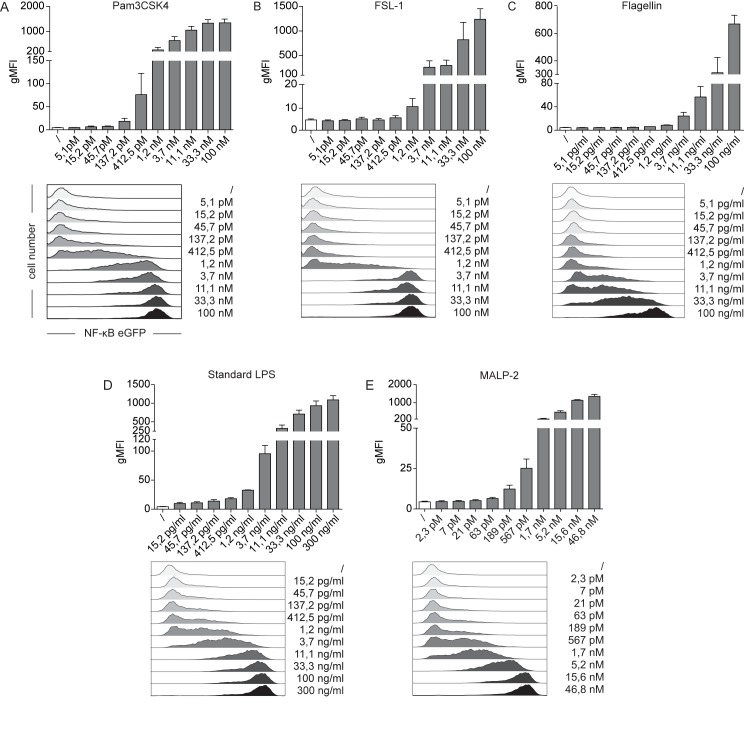
Dose-dependent response of THP-1 NF-κB-eGFP reporter cells towards specific TLR ligands. (A-E) THP-1 NF-κB-eGFP cells were incubated with increasing concentrations of Pam3CSK4, FSL-1, Flagellin, standard LPS and MALP-2 as indicated. Untreated cells served as control. After 24 h, induction of NF-κB-driven eGFP was measured by flow cytometry. Bar graphs show geometric mean of fluorescence intensity (gMFI, top panels). Mean and SE were calculated from triplicates of three independently performed experiments (n = 3). Flow cytometry histograms of a representative experiment are shown for comparison (bottom panels). Open histograms: control cells; filled histograms: TLR-activated reporter cells.

In order to further assess the sensitivity and dose-response characteristics of our THP-1 reporters we exposed the cells to serial dilutions of the TLR2 ligands Pam3CSK4 (TLR1/2), FSL-1 (TLR2/6), standard LPS (TLR2/4) and MALP-2 (TLR2/6) as well as the TLR5 ligand flagellin. Reporter gene expression was analysed 24 h later by FACS analysis. We observed dose-dependent and highly sensitive expression of eGFP in our THP-1 reporter cells, which should allow calibrating the system for quantitative measurements (Fig 2A–2E). Microbial pattern recognition by monocytes triggers the release of pro-inflammatory cytokines, like IL1-β and TNF-α. Signals emanating from the TNF-receptors and TLRs converge into the canonical pathway of NF-κB-activation [39]. TNF-α produced in response to TLR stimuli might therefore act in an autocrine feedback loop on our THP-1 reporter cells to increase NF-κB activation. To test this hypothesis, we performed blocking experiments using the clinical-grade TNF-α blocking antibody Adalimumab. Incubation of THP-1 reporter cells with TNF-α leads to a dose-dependent increase of NF-κB-eGFP expression, demonstrating the TNF-α-responsiveness of our cells (S2 Fig). Addition of the TNF-α blocking antibody could completely inhibit NF-κB-activation. We then cultured our THP-1 reporters with titrated amounts of LPS or mycoplasma supernatants in the presence or absence of TNF-α blocker. NF-κB-activity in response to TLR engagement was decreased under TNF-α blocking conditions and revealed an autocrine contribution of secreted TNF-α to total NF-κB-activity.

### High sensitivity of THP-1 NF-κB-eGFP reporter cells towards mycoplasma lipoproteins

**Fig 3:**
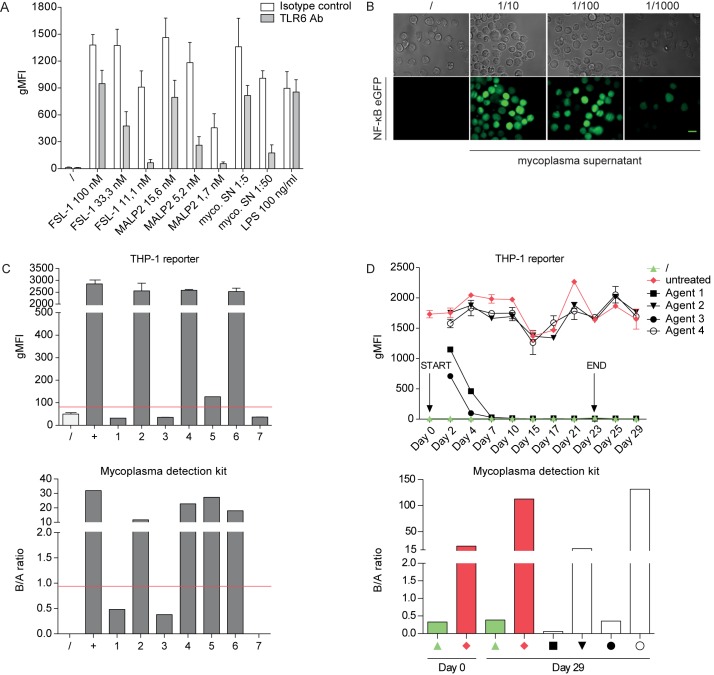
THP-1 NF-κB-eGFP reporter cells are highly sensitive towards mycoplasma lipoproteins through engagement of TLR2/6. (A) THP-1 reporter cells pre-treated with a blocking TLR6 antibody or isotype control (30 min; both used at 5 μg/mL) were incubated with the indicated concentrations of the TLR2/6 ligands FSL-1 and MALP-2 or supernatants derived from mycoplasma infected cell cultures for 24 h. Standard LPS served as a negative control. NF-κB-driven eGFP expression was assessed by flow cytometry. Bar graphs show geometric mean of fluorescence intensity (gMFI). Mean and SE were calculated from triplicates of three independently performed experiments (n = 3). (B) Fluorescent microscopy images of THP-1 reporter cells stimulated for 24 h with different dilutions of supernatants derived from mycoplasma infected cell cultures. Unstimulated cells served as negative control (left panel). Bright field images are shown for comparison (top row). Scale bar: 10 μm. (C) THP-1 NF-κB-eGFP reporter cells and a commercially available mycoplasma detection kit (MycoAlert) were probed with tissue culture supernatants from different cell sources and species: (1) mouse tail cells, (2) human mesotheliom, (3) human melanoma brain metastasis-derived cell line YDFR, (4) human LN229 glioblastoma, (5) human ovarian cancer cells, (6) COS-7 cell line and (7) human skin fibroblasts. Samples above red line are scored as positive. For the Mycoalert detection system the B/A ratio represents the ratio of the luminescence signals measured at two different time points (reading A and B). For THP-1 reporter assay mean and SE were calculated from duplicates. (D) K562 cells were infected with mycoplasma and then subjected to treatment regimens using commercially available mycoplasma removal agents (see Material and Methods). Supernatants were collected at various time points throughout the treatment course (day 2, 4, 7, 10, 15, 17, 21, 23, 25 and 29) and tested with the THP-1 NF-κB-eGFP reporter cells (upper panel). Indicated samples were also tested using the MycoAlert kit (lower panel).

Persistent mycoplasma contaminations in cell culture are a major concern in experimental research affecting the function and activity of eukaryotic cells on many levels [2]. Synthetic formulations of mycoplasma-derived diacylated lipopeptides like MALP-2 and FSL-1 are prototypic TLR2/6 ligands and our results demonstrate that our reporter cells are highly reactive to these molecules [40]. Thus we wished to explore whether our reporter cells have utility in detecting mycoplasma contaminations and probed them with supernatants from mycoplasma contaminated cell cultures. We observed that synthetic TLR2/6 ligands and infected culture supernatants both induced a strong and dose-dependent eGFP up-regulation in our THP-1 reporter cells. Moreover, a blocking antibody to TLR6 was effective in reducing reporter gene expressing elicited both by synthetic ligands and cell culture supernatants, whereas it did not affect the response to LPS (Fig 3A). This strongly indicates that TLR2/6 ligands in mycoplasma contaminated cell culture supernatants are indeed responsible for the strong reactivity of the THP-1 reporter cells. This response to the presence of mycoplasma can readily be monitored by fluorescence microscopy even when contaminated cell culture supernatants were used at high dilutions (Fig 3B). Next we wanted to assess the robustness and reliability of our reporter system by directly comparing it to a commercially available mycoplasma detection kit. Therefore we analysed cell culture supernatants derived from various cell lines that were obtained from different laboratories (S1 Table). These cell lines were put into culture and supernatants were tested in parallel using our THP-1 reporter cells and a commercial test system, which uses a bioluminescent reaction to detect mycoplasma enzyme activity (see Material and Methods). Both assays yielded identical results, which were in line with initial tests upon receipt of the cell lines (Fig 3C and S1 Table). Thus our THP-1 reporter cells allow for sensitive and cost-effective detection of mycoplasma contamination by FACS or fluorescence microscopy.

Various agents for the removal of mycoplasma contaminations in cell culture are available. They contain different classes of antibiotics and therefore differ in their mode of action. We went on to use our reporter cells to monitor the treatment of infected cell cultures using four commercially available agents (for details see Material and Methods). At the onset of the experiment, human K562 cells were deliberately infected using a generic mycoplasma supernatant. Mycoplasma removal treatment was then performed in parallel according to the manufacturer’s instructions. Supernatants collected at various time points throughout the treatment course were probed with our THP-1 reporter cells. The results demonstrated that two agents quickly removed the mycoplasma contamination in the cell cultures and supernatants obtained already after one week of treatment no longer induced reporter activity (Fig 3D, upper panel). By contrast the two other agents were ineffective as culture supernatants derived from treated cells retained their capacity to elicit strong reporter gene expression throughout the course of the treatment. Testing the cell cultures six days post treatment using a commercial detection kit fully confirmed the results obtained in our reporter experiments (Fig 3D, lower panel). These results point to a great variability in the effectiveness of commonly used mycoplasma removal regimens and highlight the importance of thorough routine testing to evaluate the success of mycoplasma treatment.

### THP-1 NF-κB-eGFP reporter cells detect mycoplasma from heat-denatured and cryo-preserved samples

**Fig 4:**
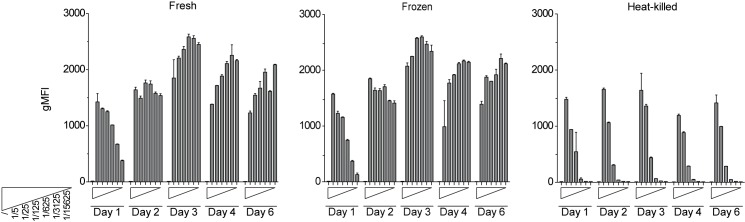
THP-1 NF-κB-eGFP reporters can detect the presence of mycoplasma in heat-denatured and cryo-preserved samples. THP-1 reporter cells were incubated with fresh (left panel), frozen (middle panel) or 95°C heat-inactivated (right panel) mycoplasma-containing tissue culture supernatants used at the indicated dilutions. NF-κB-driven eGFP expression was measured at day 1, 2, 3, 4 and 6 after the onset of the assay by flow cytometry. Bar graphs show geometric mean of fluorescence intensity (gMFI). Mean and SE were calculated from duplicates.

Standard methods for mycoplasma detection often require cellular supernatants to be analysed shortly after harvesting to preserve bacterial viability or enzyme activity. As demonstrated above, mycoplasma sensing by our THP-1 reporter cells relies on TLR2/6-mediated recognition of lipoproteins like MALP-2 and FSL-1, which are very stable molecules [41]. In principle our reporters should therefore not depend on live and or intact mycoplasma. To assess robustness of detection, we incubated our reporters with serial dilutions of fresh, frozen and 95°C heat-killed mycoplasma supernatants of identical origin and followed NF-κB-driven eGFP expression over a period of 6 days (Fig 4). Cyro-preservation of supernatants did not significantly blunt sensitivity, and even heat-inactivation retained reporter activation. Importantly, both for fresh (Fig 4, left panel) and frozen samples (Fig 4, middle panel), the serial dilution profile was quickly lost with values reaching peak responses after day two, pointing to active proliferation of mycoplasma in the reporter cell cultures. Prolonging the duration of the assay is thus an effective way to amplify and detect sub-threshold levels of mycoplasma contaminations, which might otherwise escape detection. Heat-inactivated supernatants were detected with slightly reduced sensitivity (Fig 4, right panel), while fully preserving their graded response throughout the entire six days of the assay. These data demonstrate that a heat-inactivation step can be introduced without significantly sacrificing overall sensitivity. Such pre-treatment will render supernatants biologically safe and greatly minimize the risk of mycoplasma spread within the tissue culture laboratory. Taken together, THP-1 reporter cells represent a selective and highly sensitive tool to detect mycoplasma contaminations in cell culture with considerable advantages over existing methods.

### Establishing a TLR4 sensitive THP-1 NF-κB reporter line

**Fig 5:**
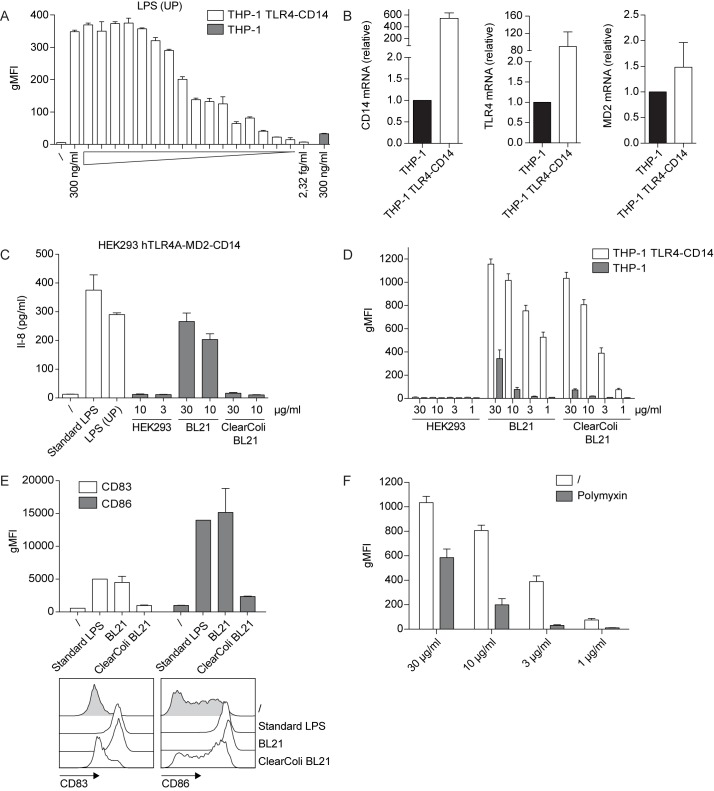
Use of THP-1 TLR4-CD14 NF-κB-eGFP reporter cells for the detection of microbial contaminations in recombinant protein preparations. (A) THP-1 TLR4-CD14 NF-κB-eGFP reporter cells were incubated with the indicated serial dilutions of ultrapure LPS for 24 h. NF-κB-driven eGFP expression was assessed by flow cytometry. Bar graphs show geometric mean of fluorescence intensity (gMFI). eGFP expression of the parental THP-1 reporter cells treated with 300 ng/ml ultrapure LPS is shown for comparison (grey bar). (B) mRNA expression of TLR4, CD14 and MD2 was measured in parental and TLR4-CD14 THP-1 reporter cells by real-time qPCR. Data are presented as mean ± SEM of expression values normalized against the housekeeping gene GAPDH (n = 4). (C) Recombinant human split product C4dg produced in three different expression systems (HEK293-6E, standard *E*. *coli* BL21 and *E*. *coli* ClearColi BL21) was tested for TLR4-agonist contaminations using HEK293 hTLR4A-MD2-CD14 cells. Following 24 h of incubation the IL-8 content in the culture supernatants was measured by ELISA. (D) Parental and TLR4-CD14 THP-1 reporters were incubated with the protein preparations described in (C) and NF-κB-driven eGFP expression was assessed by flow cytometry 24 h later. Bar graphs show geometric mean of fluorescence intensity (gMFI). Mean and SE were calculated from duplicates of four independently performed experiments (n = 4) (E) Immature human moDCs were incubated with standard LPS, *E*. *coli* BL21 or *E*. *coli* ClearColi BL21 expressed C4dg protein at the indicated concentrations for 24 h or were left untreated. Expression of maturation markers CD83 and CD86 was assessed by flow cytometry. (F) *E*. *coli* ClearColi BL21-expressed C4dg protein was subjected to a single round of bulk chromatography using a polymyxin resin (see Material and Methods). Samples before and after chromatography were tested using the THP-1 TLR4-CD14 reporters. Mean and SE were calculated from duplicates of four independently performed experiments (n = 4).

As shown above, our THP-1 NF-κB reporter cells are unresponsive towards TLR4 ligands, like highly pure LPS. LPS sensing is unique in that TLR4 relies on the accessory molecule MD2 to capture its ligand LPS. Secreted LPS binding protein (LBP) and surface or soluble CD14 assist in the transfer of LPS from bacterial membranes to TLR4-MD2. The TLR4-MD2-LPS complex dimerizes, thereby initiating signalling from the intracellular TIR domains contained in TLR4 [16]. A possible explanation for the observed unresponsiveness of our THP-1 reporter cells towards ultrapure LPS is the lack of TLR4 and/or accessory molecule expression like CD14. Thus, in an effort to furnish our reporter cells with reactivity to TLR4 ligands, we introduced TLR4 and the accessory molecules CD14 and MD2 into our THP-1 reporter cells by retroviral transduction. Following cell sorting and cloning by limiting dilution, a single cell clone with high sensitivity towards highly pure LPS was obtained (Fig 5A). In comparison to the parental THP-1 reporter cells, this clone (THP-1 TLR4-CD14 NF-κB-eGFP hereafter) exhibited a significant increase of CD14 and TLR4 expression as shown by qPCR analysis (Fig 5B).

To assess the sensitivity of our THP-1 TLR4-CD14 reporter cells to microbial contaminations in more complex biological samples, we focussed on a recombinant protein produced in different expression systems. Human complement split product C4dg, with a C-terminal 6xHIS Tag (C4dg-HIS) was chosen as model protein. Three different host cells were used for protein expression: Human embryonic kidney cells subline 293-6E (HEK293-6E), standard *E*. *coli* BL21 and *E*. *coli* ClearColi BL21. A highly efficient serum-free mammalian expression system based on the HEK293-6E cell line has been described in detail [30]. The recently developed endotoxin-free *E*. *coli* strain ClearColi BL21 is characterized by modifications in the LPS structure to disable TLR4 triggering [42, 43]. Purification of recombinant protein from all three hosts was performed by cobalt-based HisTALON immobilized metal affinity chromatography following identical workflows. First, we assessed the degree of LPS contamination by using HEK293 hTLR4A-MD2-CD14 cells, which produce Interleukin-8 in response to TLR4 engagement. We could indeed confirm that *E*. *coli* ClearColi BL21 and HEK293-6E protein preparations are free of TLR4-reactive impurities, whereas *E*. *coli* BL21 expressed C4dg induced strong TLR4 triggering (Fig 5C). However, when using THP-1 TLR4-CD14 reporter cells, both proteins from *E*. *coli* BL21 and *E*. *coli* ClearColi BL21 elicited high NF-κB-driven eGFP induction, while no response was obtained with mammalian-expressed protein (Fig 5D). In comparison, parental THP-1 reporter cells showed only weak reactivity towards proteins derived from both bacterial strains. CD14 is a well-documented amplifier of non-TLR4-mediated signals [44–46]. Thus, the heightened responsiveness towards ClearColi BL21 protein, as seen in our THP-1 TLR4-CD14 reporter cells, may be attributed to increased CD14 expression (Fig 5B and 5D). ClearColi expressed protein also induced upregulation of maturation markers on human moDCs albeit at much lower level than protein expressed in standard BL21 (Fig 5E). We next wanted to assess whether such residual impurities could be removed by standard endotoxin removal regimens. We chose an affinity matrix based on polymyxin B, a cationic cyclic antibiotic isolated from Bacillus polymyxa, which displays high affinity for bacterial lipopolysaccharides [47]. Recombinant C4dg from ClearColi BL21 was subjected to treatment with a polymyxin B resin and then tested on THP-1 TLR4-CD14 reporter cells (Fig 5F). Despite the absence of classical LPS contamination, polymyxin-treatment of ClearColi BL21-expressed protein led to a strong reduction in THP-1 reporter cell activation. Previous studies using HEK293 reporter cells expressing human TLR2 have shown that polymyxin B can indeed supress non-TLR4 impurities in LPS preparations [48]. Our findings highlight the importance to use test-systems sensitive towards a broad array of microbial contaminations. We believe that the newly engineered THP-1 TLR4-CD14 reporter cell line represents such a system combining broad specificity, high sensitivity and ease of use.

## Discussion

Human Toll-like receptors (TLRs) play a central role in the first-line defence mechanisms of innate immunity mediating recognition of conserved pathogen-associated molecular patterns (PAMPs). TLRs have evolved to monitor both the extracellular and endosomal/lysosomal space for the presence of microbial danger. Recent progress in our understanding of TLR biology has led to the identification of a plethora of microbial ligands for TLRs [21]. The activation of the transcription factor NF-κB is a major consequence of TLR-engagement by PAMPs. NF-κB induces the release of cytokines and chemokines and thus acts as a key mediator of inflammatory responses, which are mandatory to control and eventually eliminate infections. For the current study, we aimed to exploit the exquisite sensitivity and specificity of the TLR signalling machinery for the development of a cell-based, fluorescent reporter system. THP-1, a human monocytic cell line expressing a wide range of TLRs [33], was equipped with an NF-κB-driven reporter construct we have described recently [26, 31, 49]. Our reporter cells are amenable to high-throughput analysis of NF-κB-activity by flow cytometry. Importantly, in comparison to plate-based readout systems, acquisition by flow cytometry delivers information on cell viability, which is a relevant parameter when testing cytotoxic pharmacological substances.

We observed that the resulting THP-1 NF-κB-eGFP reporter cell line is highly reactive to ligands for TLR2 heterodimers. Since mycoplasmas are abundant in TLR2/6 agonists it was explored whether these cells are useful for the detection of mycoplasma in cell culture samples. Mycoplasma contamination is a common and persistent problem in cell cultures, with up to 35% of continuously cultured cell lines testing positive [50]. The presence of these bacteria can lead to significant disturbances in experimental research due to alterations in gene expression, chromosomal aberrations and changes in membrane composition [2]. Additionally, chronic engagement of the TLR2 pathway may lead to artefactual results in cells of the myeloid linage [51, 52]. Lack of a rigid cell wall makes beta-lactam antibiotics, which are commonly used as tissue-culture supplement, ineffective against mycoplasma. Moreover their small size and pleomorphic shape allows mycoplasma to pass through standard sterile filtration units [2]. Thus highly sensitive, standardized and cost-effective detection methods as well as efficient removal regimens for mycoplasma contamination in cultured cells are of crucial importance. Most mycoplasma tests either rely on PCR, qPCR, biochemical analysis with labelled probes or lengthy agar and broth culture methods [53]. Since the use of these techniques is often expensive and time consuming, there is demand for alternative testing methods. We demonstrate here that THP-1 NF-κB-eGFP reporter cells reliably detect mycoplasma contaminations in cell culture supernatants from different sources and species. Moreover, our results indicate that TLR6 engagement is the basis of mycoplasma sensing by these cells. TLRs generally recognize highly conserved structures, which play vital roles in the biology of microorganisms and it thus can be expected that THP-1 reporter cells will detect contaminations caused by all strains of mycoplasma. Moreover, mycoplasma lipopeptides are stable compounds and as a consequence, THP-1 reporter cells detect the presence of mycoplasma in heat-denatured and cryo-stored samples with high sensitivity. Implementing a heat-inactivation step for biological samples prior to testing will greatly minimize the risk of mycoplasma spread within the tissue culture laboratory without sacrificing detection sensitivity. We also employed our THP-1 reporter cells to follow the efficacy of mycoplasma decontamination using four different commercially available antibiotics. To our surprise, successful removal of mycoplasma contaminations was achieved by only two of the four tested treatment compounds. Chronic infections with Plasmocin-resistant mycoplasma has been reported [54]. The authors could clear such established infections by the use of BM-cyclin, which we also found to be efficient in eradicating mycoplasma. Taken together, our THP-1-based reporter cell system is ideally suited for routine testing of mycoplasma contaminations in tissue culture supernatants. It is highly sensitive and cost-effective and can be performed on a high-throughput level without the need for additional reagents.

Detection and quantification of endotoxins in biological samples, especially recombinant proteins expressed in bacterial hosts, was another obvious and highly relevant application for our reporter system. Recombinant protein expression in *Escherichia coli (E*.*coli)* is widely used in basic research and a third of all approved recombinant therapeutics is produced in this host system [55]. Major advantages include ease of handling, cost-effectiveness and high yields. However, isolation of recombinant proteins from bacterial homogenates bears the risk of contamination with cell wall components or lipids. Presence of endotoxin in recombinant protein preparations can perturb experimental results and preclude in-vivo applications. Indeed, sensitive primary cells like CD1c+ dendritic cells were demonstrated to respond even to minimal amounts of LPS, as present in commercial protein preparation of high purity [56]. Thus thorough downstream endotoxin-removal protocols need to be implemented, combined with ultra-sensitive testing methods. The current gold standard for gram-negative endotoxin testing in the pharmaceutical industry is the Limulus amebocyte lysate (LAL) test, which relies on an extract of blood cells from the horseshoe crab, *Limulus polyphemus* [1]. Although crabs are released alive after a fraction of their blood is collected, significant post-bleeding mortality rates of 5–30% have been reported [57]. In light of the growing demand for reliable endotoxin tests, it is therefore highly desirable to find in-vitro substitutes for the LAL test. Despite being of monocytic origin, our THP-1 reporter cells initially failed to respond to ultrapure LPS, which represents a TLR4-only ligand. TLR4 triggering by LPS relies on the cofactors CD14 and MD2 [16]. By retrovirally introducing TLR4, CD14 and MD2, we broadened the target range of our THP-1 reporters towards TLR4 PAMPs and rendered the resulting cells ultra-sensitive for a diversity of endotoxin contaminations. Consequently, we employed our THP-1 TLR4-CD14 reporters to assess PAMP contamination of a recombinant protein produced in three different hosts: *E*. *coli* BL21, *E*. *coli* ClearColi BL21 and mammalian HEK293-6E cells. ClearColi BL21 is a novel strain of *E*. *coli* BL21, which has been genetically modified to express an altered form of the LPS molecule, missing two out of six acyl chains thereby effectively abolishing TLR4 triggering [42]. Despite the clear absence of TLR4 reactivity, ClearColi-derived proteins still induced strong activation of THP-1 TLR4-CD14 reporter cells, indicating considerable contamination with bacterial PAMPs. ClearColi expressed proteins also induced activation of moDCs but to a much lower extent compared to the THP-1 TLR4-CD14 reporter cells. This result is consistent with the much lower sensitivity of moDCs towards TLR2 ligands, which are the likely PAMPs contained in ClearColi-expressed proteins.

Polymyxin-B treatment of ClearColi-derived proteins led to a significant reduction in THP-1 TLR4-CD14 reporter activity. This is an unexpected finding, since the only LPS related molecule expressed in ClearColi BL21 is the tetraacylated endotoxin precursor lipid IV_A_, which does not trigger TLR4 responses (Fig 5C) [42]. However, *E*. *coli* LPS preparations were reported to contain LPS-mimetic TLR2-stimulatory substances other than lipopeptides or peptidoglycans [58]. Additionally, polymyxin B has been shown to inhibit TLR2 stimulation via endotoxin proteins only when they were physically associated with LPS [34, 48]. Therefore, a possible explanation for our findings is that, like intact LPS, lipid IV_A_ binds to polymyxin B. Lipid IV_A_ and closely associated endotoxin proteins are thereby removed from the protein preparation, resulting in reduced TLR2 reactivity. Our findings are relevant for experimental research relying on *E*. *coli*-expressed recombinant proteins and demonstrate the importance to test for contaminations beyond the TLR4 spectrum.

Overall, by harnessing the evolutionary-conserved sensitivity of the TLR system and combining it with our recently described fluorescent reporter technology [26], we have generated a highly sensitive and versatile cellular test platform for microbial contaminants. We have successfully applied our THP-1 NF-κB-eGFP reporter cells to the detection of mycoplasma and endotoxin in biological samples. THP-1 and murine RAW macrophage-based NF-κB reporters, employing secreted alkaline phosphatase as readout, have been described previously [59, 60]. These systems require additional handling steps and non-standard reagents. In comparison, our cell-intrinsic fluorescent reporter system is unique in that it allows cost effective high-throughput measurements with minimal hands-on time on standard flow cytometers without the need for costly reagents.

## Supporting information

S1 Table Cell culture supernatants. Tissue culture supernatants from different cell sources and species were tested for mycoplasma lipoprotein contaminations. The results for three different detection methods are shown: PCR-based technique, MycoAlert kit and THP-1 reporter assay.
(PDF)

S1 Fig Generation of the monocytic THP-1 reporter cell line (THP-1 NF-κB-eGFP). (A) Schematic illustration of the reporter construct encoding NF-κB-eGFP with restriction enzyme recognition sites. MP: minimal promoter. (B) The retroviral NF-κB-driven eGFP reporter construct was introduced into THP-1 cells and resting eGFP-low expressing cells were sorted. From this cell pool single cell clones were established by limiting dilution culturing. A cell clone that was negative in a resting state and strongly expressed the reporter gene upon activation with PMA and Ionomycin was selected for further testing.
(EPS)

S2 Fig Effect of a TNF-α blocking antibody on activated THP-1 reporter cells. THP-1 reporter cells were incubated with the monoclonal TNF-α blocking antibody Adalimumab (10 μg/ml) and with the indicated concentrations of LPS, recombinant TNF-α or mycoplasma supernatants. NF-κB-driven eGFP expression was assessed by flow cytometry. Bar graphs show geometric mean of fluorescence intensity (gMFI). Mean and SE were calculated from triplicates of three independently performed experiments (n = 3).
(EPS)
