# Supplementary material for: A human monocytic NF-κB fluorescent reporter cell line for detection of microbial contaminants in biological samples
Source: PLoS One. 2017 May 24;12(5):e0178220. doi: 10.1371/journal.pone.0178220 (PMC5443541; doi:10.1371/journal.pone.0178220)
Supplement: S1 Table — Tissue culture supernatants from different cell sources and species were tested for mycoplasma lipoprotein contaminations. The results for three different detection methods are shown: PCR-based technique, MycoAlert kit and THP-1 reporter assay. (PDF) [file pone.0178220.s001.pdf]

Nr.	Cell line	PCR	Mycoalert	Reporter assay
**1**	Mouse tail cells	−	−	−
**2**	Human Mesotheliom	+	+	+
**3**	Human melanoma brain metastasis-derived cell line YDFR	−	−	−
**4**	LN229 glioblastoma	+	+	+
**5**	Human ovarian cancer cells	−	−	−
**6**	COS-7 cell line	+	+	+
**7**	Human skin fibroblasts	−	−	−
